# A novel associative memory model based on semi-tensor product (STP)

**DOI:** 10.3389/fncom.2024.1384924

**Published:** 2024-03-19

**Authors:** Yanfang Hou, Hui Tian, Chengmao Wang

**Affiliations:** ^1^School of Electrical and Electronic Engineering, Chongqing University of Technology, Chongqing, China; ^2^School of Automation, Chongqing University of Posts and Telecommunications, Chongqiong, China

**Keywords:** associative memory model, discrete Hopfield neural network (DHNN), information storage capacity, semi-tensor product (STP) of matrices, associative memory matrix

## Abstract

A good intelligent learning model is the key to complete recognition of scene information and accurate recognition of specific targets in intelligent unmanned system. This study proposes a new associative memory model based on the semi-tensor product (STP) of matrices, to address the problems of information storage capacity and association. First, some preliminaries are introduced to facilitate modeling, and the problem of information storage capacity in the application of discrete Hopfield neural network (DHNN) to associative memory is pointed out. Second, learning modes are equivalently converted into their algebraic forms by using STP. A memory matrix is constructed to accurately remember these learning modes. Furthermore, an algorithm for updating the memory matrix is developed to improve the association ability of the model. And another algorithm is provided to show how our model learns and associates. Finally, some examples are given to demonstrate the effectiveness and advantages of our results. Compared with mainstream DHNNs, our model can remember learning modes more accurately with fewer nodes.

## 1 Introduction

Under complex circumstances, autonomous perception and scene understanding of the environment are a prerequisite for intelligent unmanned systems to operate autonomously (Gonzalez-Jorge et al., 2017; Muniraj and Farhood, 2019). It directly affects whether the task can be successfully completed. By utilizing multiple sensors to collaborate with each other, the environmental feature information is extracted, and a sufficient number of learning datasets with precise calibration are established (Balestrieri et al., 2021). Then, building an intelligent learning model (Jafari and Xu, 2018; Eski and Kus, 2019; Guo et al., 2022; Kang et al., 2022) can achieve complete recognition of scene information and accurate recognition of designated targets by intelligent unmanned systems.

There are many intelligent learning models, such as neural networks (Ding et al., 2019; Pasa et al., 2022; García-Treviño et al., 2024) and fuzzy control (Zhang et al., 2021; Su et al., 2023), etc. They are effective in modeling and dealing with many non-linear systems. The discrete Hopfield neural network (DHNN) is a classical neural network (Tank and Hopfield, 1986; Kobayashi, 2019a). It adopts a fully interconnected and completely feedback structure. Therefore, the output of any neuron is fed back to all neurons as inputs through connection weights. Its purpose is to enable the output of any neuron to be controlled by the output of all neurons so that the outputs of all neurons can be mutually constrained. Associative memory is the primary function of DHNN. The working process is divided into two stages: memory and association. In the memory stage, through design or learning of network weights, the network has several equilibrium states, which are also known as attractors. These attractors are the extreme points of the energy function of the DHNN. The memory process is to store those modes that need to be remembered as network attractors. The associative stage is such a process that the associative memory network reaches a stable state from a given state through the evolution of dynamics, i.e., converging to the attractors and recalling stored modes.

The number of attractors represents the memory capacity or storage capacity of the network. It is actually the maximum number of non-interfering modes stored in the network under a certain association error tolerance. The more attractors a network has, the larger the information storage capacity is and the stronger the associative ability is. In fact, when using DHNN for associative memory, it is constrained by memory capacity and sample differences (Kobayashi, 2019b). If there are too many samples to memorize the network may converge to a pseudo mode that is different from any learning mode. Moreover, when the network scale is fixed, the number of modes that can be remembered is very limited. In general, the maximum number of modes that a network can store is called the network capacity. It is related to size, algorithm, and vector distribution of memory modes of the network. As the number of memory modes increases, some memory modes intersect with each other. When the number of modes exceeds the network capacity, the network not only gradually forgets the modes previously memorized but also cannot remember the new modes. For a DHNN with *n* neurons, the maximum number of modes that can be remembered is 0.15*n* by appropriately selecting the connection weight matrix. It indicates that when the network size is fixed, the more the modes to be remembered are, the greater the likelihood of making mistakes during the association is. On the contrary, the lower the allowable error is, the smaller the information storage capacity is.

To solve the problem of information storage capacity, this study proposes a new associative memory model. The main mathematical tool is the semi-tensor product (STP) of matrices, which is proposed and developed by Cheng'group (Cheng and Qi, 2009). STP extends ordinary matrix multiplication to a general case, where the number of columns in the front matrix is not required to equal to that of rows in the back matrix. It not only retains the basic properties of the ordinary matrix multiplication but also has some special properties, such as pseudo commutativity. After development in the past two decades, STP has made significant progress in both theory and application, including logical control systems (Zhang et al., 2016; Tian et al., 2017; Tian and Hou, 2019), finite game (Zheng et al., 2022; Hou and Tian, 2023), and finite automata (Zhang et al., 2020; Yan et al., 2023), and so on.

The main contributions are summarized as follows:

1. A new associative memory model is proposed. Compared with the mainstream DHNNs, the new model can accurately remember more learning modes with fewer nodes.
2. Unlike most existing models that perform mode recognition after learning, our model automatically learns when needed.
3. The accuracy of mode recognition can be controlled effectively using our recognition model.

The rest of the study is organized as follows. In Section 2, some preliminaries are introduced, including DHNN, outer product method, and STP. In addition, the problem of information storage capacity is pointed out. Section 3 proposes a new memory model and its update algorithm. The learning and association process of the new model is provided in another algorithm. Section 4 gives an illustrative example, which is followed by a brief conclusion mentioned in Section 5.

## 2 Materials and methods

### 2.1 Preliminaries

#### 2.1.1 Notations

For ease of expression, some notations are first introduced.

- ℝ^*n*^: the set of all *n*-dimensional real vectors;
- 𝕄_*m*×*n*_: the set of *m* × *n* dimensional real matrices;
- *Col*_*i*_(*M*) (*Row*_*i*_(*M*)): the *i*th column (row) of matrix *M*;
- ***0***: a zero column vector with a appropriate dimension;
- $\delta_{n}^{i}:=Col_{i}\left(I_{n}\right)$, the *i*th column of *n* × *n* dimensional identity matrix *I*_*n*_;
- $\Delta_{n}:=Col\left(I_{n}\right)=\left\{\delta_{n}^{i}|i=1,2,\cdots \;,n\right\}$, the set of all columns of *I*_*n*_;
- $r={\left[r_{1},\cdots \;,r_{n}\right]}^{T}\in {\mathbb{R}}^{n}$ is a probabilistic vector, if *r*_*i*_ ≥ 0, *i* = 1⋯ , *n*, and $\sum_{i=1}^{n}r_{i}=1$.

#### 2.1.2 Discrete Hopfield neural network

Discrete Hopfield Neural Network (DHNN) is a type of binary neural network. Each neuron's output only takes two states 1 and –1, representing activation and inhibition, respectively. It mainly has the following characteristics:

1. Each unit has no self feedback (no connection to itself). That is, *w*_*ii*_ = 0.
2. The connection weights between units are symmetrical, i.e *w*_*ij*_ = *w*_*ji*_, ∀*i* ≠ *j*.

For a DHNN with *n* binary units, it is easy to know that the cardinality of its state space is 2^*n*^.

The associative memory is an important function of DHNN. The core of implementing associative memory is to design a set of appropriate network connection weights and thresholds, based on energy extreme points (also known as learning modes). There are many design methods and learning algorithms for connection weight of DHNN, such as outer product method, projection learning rule, pseudo inverse method, and eigen structure method, etc. However, the most commonly used method is the outer product method that is based on Hebb learning rules. We recall it in the following.

#### 2.1.3 Semi-tensor product of matrices

Since the main mathematical tool used in this study is STP, it is necessary to recall it here.

Definition 1. Cheng and Qi (2010) Let *A* ∈ 𝕄_*m*×*n*_, *B* ∈ 𝕄_*p*×*q*_, and denote the least common multiplier *lcm*(*n, p*) of *n* and *p* by *r*. Then, the STP of *A* and *B* is


$$A⋉B:=\left(A⊗I_{\frac{r}{n}}\right)\left(B⊗I_{\frac{r}{p}}\right)\in {𝕄}_{\frac{mr}{n}\times \frac{qr}{p}},$$

where ⊗ is the Kronecker product of matrices.

It is noted that STP has properties similar to those of the ordinary matrix products. Especially, when *n* = *p*, STP happens to be the ordinary matrix product. Therefore, STP is generalization of the ordinary matrix product. Throughout this study, the matrix products are STP and the symbol ⋉ is usually omitted.

Here, it is necessary to recall an important property. Readers refer to Cheng et al. (2011) for more details.

Let $x={\left(x_{1},x_{2},\cdots \;,x_{m}\right)}^{T}\in {\mathbb{R}}^{m}$, $y={\left(y_{1},y_{2},\cdots \;,y_{n}\right)}^{T}\in {\mathbb{R}}^{n}$. Then


$$x⋉y={\left(x_{1}y_{1},\cdots \;,x_{1}y_{n},\cdots \;,x_{m}y_{1},\cdots \;,x_{m}y_{n}\right)}^{T}\in {\mathbb{R}}^{mn}.$$

### 2.2 Problem analysis

Although DHNN has strong associative memory function, it also has some shortcomings. They are listed as follows:

- The limitation of memory capacity.
- When the memory modes are relatively close, the mode associated or recovered by the network may not be the closest to the input one.
- In some cases, the mode recalled by the network is not any of the memory modes. It leads to falling into a “pseudo state.”

Next, an example will be used to illustrate these problems faced when applying DHNN to associative memory.

Example 1. Consider a DHNN with five nodes, whose learning modes are *X*^1^ = (1, 1, −1, 1, 1)^*T*^, *X*^2^ = (1, −1, 1, −1, −1)^*T*^, *X*^3^ = (−1, 1, 1, −1, 1)^*T*^, and *X*^4^ = (−1, −1, −1, 1, 1)^*T*^. Calculate its connection weight matrix *W* and verify its memory ability.

First, according to Equation 1 in Algorithm 1, the connection weight matrix *W* can be computed as follows:


$$\begin{array}{l} W=X^{1}{\left(X^{1}\right)}^{T}+X^{2}{\left(X^{2}\right)}^{T}+X^{3}{\left(X^{3}\right)}^{T}+X^{4}{\left(X^{4}\right)}^{T}-4I\\ \;=\left(\begin{array}{c} 1\\ 1\\ -1\\ 1\\ 1 \end{array}\right)\left(\begin{array}{ccccc} 1 & 1 & -1 & 1 & 1 \end{array}\right)+\left(\begin{array}{c} 1\\ -1\\ 1\\ -1\\ -1 \end{array}\right)\left(\begin{array}{ccccc} 1 & -1 & 1 & -1 & -1 \end{array}\right)+\\ \;\left(\begin{array}{c} -1\\ 1\\ 1\\ -1\\ 1 \end{array}\right)\left(\begin{array}{ccccc} -1 & 1 & 1 & -1 & 1 \end{array}\right)\\ \;+\left(\begin{array}{c} -1\\ -1\\ -1\\ 1\\ 1 \end{array}\right)\left(\begin{array}{ccccc} -1 & -1 & -1 & 1 & 1 \end{array}\right)-4\left(\begin{array}{ccccc} 1 & 0 & 0 & 0 & 0\\ 0 & 1 & 0 & 0 & 0\\ 0 & 0 & 1 & 0 & 0\\ 0 & 0 & 0 & 1 & 0\\ 0 & 0 & 0 & 0 & 1 \end{array}\right)\\ \;=\left(\begin{array}{ccccc} 1 & 1 & -1 & 1 & 1\\ 1 & 1 & -1 & 1 & 1\\ -1 & -1 & 1 & -1 & -1\\ 1 & 1 & -1 & 1 & 1\\ 1 & 1 & -1 & 1 & 1 \end{array}\right)+\left(\begin{array}{ccccc} 1 & -1 & 1 & -1 & -1\\ -1 & 1 & -1 & 1 & 1\\ 1 & -1 & 1 & -1 & -1\\ -1 & 1 & -1 & 1 & 1\\ -1 & 1 & -1 & 1 & 1 \end{array}\right)+\\ \;\left(\begin{array}{ccccc} 1 & -1 & -1 & 1 & -1\\ -1 & 1 & 1 & -1 & 1\\ -1 & 1 & 1 & -1 & 1\\ 1 & -1 & -1 & 1 & -1\\ -1 & 1 & 1 & -1 & 1 \end{array}\right)\\ \;+\left(\begin{array}{ccccc} 1 & 1 & 1 & -1 & -1\\ 1 & 1 & 1 & -1 & -1\\ 1 & 1 & 1 & -1 & -1\\ -1 & -1 & -1 & 1 & 1\\ -1 & -1 & -1 & 1 & 1 \end{array}\right)-\left(\begin{array}{ccccc} 4 & 0 & 0 & 0 & 0\\ 0 & 4 & 0 & 0 & 0\\ 0 & 0 & 4 & 0 & 0\\ 0 & 0 & 0 & 4 & 0\\ 0 & 0 & 0 & 0 & 4 \end{array}\right)\\ \;=\left(\begin{array}{ccccc} 0 & 0 & 0 & 0 & -2\\ 0 & 0 & 0 & 0 & 2\\ 0 & 0 & 0 & -4 & -2\\ 0 & 0 & -4 & 0 & 2\\ -2 & 2 & -2 & 2 & 0 \end{array}\right). \end{array}$$

Next, we use *W* that is obtained above to verify the memory ability of the network.


$$\begin{array}{l} \text{sgn}(WX^{1})=\text{sgn}(\left(\begin{array}{ccccc} 0 & 0 & 0 & 0 & -2\\ 0 & 0 & 0 & 0 & 2\\ 0 & 0 & 0 & -4 & -2\\ 0 & 0 & -4 & 0 & 2\\ -2 & 2 & -2 & 2 & 0 \end{array}\right)\left(\begin{array}{c} 1\\ 1\\ -1\\ 1\\ 1 \end{array}\right))\\ \;=\text{sgn}({\left(\begin{array}{ccccc} -2 & 2 & -6 & 6 & 4 \end{array}\right)}^{T})={\left(\begin{array}{ccccc} -1 & 1 & -1 & 1 & 1 \end{array}\right)}^{T}\neq X^{1}. \end{array}$$

$$\begin{array}{l} \text{sgn}(WX^{2})=\text{sgn}(\left(\begin{array}{ccccc} 0 & 0 & 0 & 0 & -2\\ 0 & 0 & 0 & 0 & 2\\ 0 & 0 & 0 & -4 & -2\\ 0 & 0 & -4 & 0 & 2\\ -2 & 2 & -2 & 2 & 0 \end{array}\right)\left(\begin{array}{c} 1\\ -1\\ 1\\ -1\\ -1 \end{array}\right))\\ \;=\text{sgn}({\left(\begin{array}{ccccc} 2 & -2 & 6 & -6 & -8 \end{array}\right)}^{T})=\\ \;{\left(\begin{array}{ccccc} 1 & -1 & 1 & -1 & -1 \end{array}\right)}^{T}=X^{2}. \end{array}$$

$$\begin{array}{l} \text{sgn}(WX^{3})=\text{sgn}(\left(\begin{array}{ccccc} 0 & 0 & 0 & 0 & -2\\ 0 & 0 & 0 & 0 & 2\\ 0 & 0 & 0 & -4 & -2\\ 0 & 0 & -4 & 0 & 2\\ -2 & 2 & -2 & 2 & 0 \end{array}\right)\left(\begin{array}{c} -1\\ 1\\ 1\\ -1\\ 1 \end{array}\right))\\ \;=\text{sgn}({\left(\begin{array}{ccccc} -2 & 2 & 2 & -2 & 0 \end{array}\right)}^{T})=\\ \;{\left(\begin{array}{ccccc} -1 & 1 & 1 & -1 & -1 \end{array}\right)}^{T}\neq X^{3}. \end{array}$$

$$\begin{array}{l} \text{sgn}(WX^{4})=\text{sgn}(\left(\begin{array}{ccccc} 0 & 0 & 0 & 0 & -2\\ 0 & 0 & 0 & 0 & 2\\ 0 & 0 & 0 & -4 & -2\\ 0 & 0 & -4 & 0 & 2\\ -2 & 2 & -2 & 2 & 0 \end{array}\right)\left(\begin{array}{c} -1\\ -1\\ -1\\ 1\\ 1 \end{array}\right))\\ \;=\text{sgn}({\left(\begin{array}{ccccc} -2 & 2 & -6 & 6 & 4 \end{array}\right)}^{T})=\\ \;{\left(\begin{array}{ccccc} -1 & 1 & -1 & 1 & 1 \end{array}\right)}^{T}\neq X^{4}. \end{array}$$

**Algorithm 1:** Outer product method based on Hebb learning rules.

*Assume that the network has* *n* *nodes and the connection weight matrix of the network is* *W*. *If the network processes* *m* *pairwise orthogonal pattern samples, i.e., the learning task set of the network is* $X^{k}={\left(x_{1}^{k},x_{2}^{k},\cdots \;,x_{n}^{k}\right)}^{T},k=1,2,\cdots \;,m$, *W* *is calculated as follows:*
(1) $$\begin{array}{l} W=\alpha \overset{m}{\underset{k=1}{\sum }}\left[X^{k}{\left(X^{k}\right)}^{T}-I\right], \end{array}$$
where α is a positive constant.

From the above results, it can be observed that the network cannot perform normal associative memory on learning modes *X*^1^, *X*^3^, and *X*^4^ because the input modes *X*^1^, *X*^2^, *X*^3^, and *X*^4^ are non-orthogonal. When the modes to be memorized are similar, it is easy for them to intersect with each other. The network cannot fully distinguish these modes. Moreover, errors may still occur, even if a memorized mode is used as the object to be recognized. In fact, for non-orthogonal modes of a DHNN with *n* neurons, the information storage capacity is generally 0.13*n*–0.15*n*. When the number of storage modes exceeds 0.15*n*, the associative memory of the network may go wrong.

## 3 Results

### 3.1 A new memory model

For any learning mode $X^{k}={\left(l_{1},l_{2},\cdots \;,l_{n}\right)}^{T}$ of a DHNN with *n* nodes, we first transform it into an equivalent vector $Y^{k}={\left(k_{1},k_{2},\cdots \;,k_{n}\right)}^{T}$, where


$$k_{i}=\{\begin{array}{c} l_{i},\text{if }l_{i}=1,\\ 0,\text{otherwise}. \end{array}$$

Next, we continue to give the algebraic form of *Y*^*k*^. $\delta_{2}^{2-i}$ is identified with *i*, *i* = 0, 1. Then, ${\left(k_{1},k_{2},\cdots \;,k_{n}\right)}^{T}$ can be replaced by ${\left(\delta_{2}^{2-k_{1}},\delta_{2}^{2-k_{2}},\cdots \;,\delta_{2}^{2-k_{n}}\right)}^{T}$. From the study by Cheng et al. (2011), ${\left(\delta_{2}^{2-k_{1}},\delta_{2}^{2-k_{2}},\cdots \;,\delta_{2}^{2-k_{n}}\right)}^{T}$ is equivalent to $\delta_{2^{n}}^{r_{k}}$, where $r_{k}=\left(1-k_{1}\right)2^{n-1}+\left(1-k_{2}\right)2^{n-2}+\cdots +\left(1-k_{n-1}\right)2+\left(2-k_{n}\right)$:


$$Y^{k}={\left(k_{1},k_{2},\cdots \;,k_{n}\right)}^{T}\sim \delta_{2}^{2-k_{1}}\delta_{2}^{2-k_{2}}\cdots \delta_{2}^{2-k_{n}}=\delta_{2^{n}}^{r_{k}},$$

where $\delta_{2^{n}}^{r_{k}}$ is called the algebraic form of the learning mode *Y*^*k*^.

In the following, we give a new memory model based on algebraic forms of learning modes.

Theorem 1. Assume that *n*-dimensional modes *Y*^*k*^, *k* = 1, 2, ⋯ , *m*, are required to be memorized, whose algebraic forms are $\delta_{2^{n}}^{r_{k}}$, respectively. A memory matrix is designed as follows:


(2) $$\begin{array}{l} L=\overset{m}{\underset{k=1}{\sum }}\delta_{2^{n}}^{r_{k}}{\left(\delta_{2^{n}}^{r_{k}}\right)}^{T}. \end{array}$$

Then, all modes *Y*^*k*^ are memorized by *L* and fully recognized.

PROOF. For any mode *Y*^*i*^, its algebraic form is $\delta_{2^{n}}^{r_{i}}$. Then,


$$L\delta_{2^{n}}^{r_{i}}=\overset{m}{\underset{k=1}{\sum }}\delta_{2^{n}}^{r_{k}}{\left(\delta_{2^{n}}^{r_{k}}\right)}^{T}\delta_{2^{n}}^{r_{i}}.$$

Since ${\left(\delta_{2^{n}}^{r_{i}}\right)}^{T}\delta_{2^{n}}^{r_{i}}=1$ and ${\left(\delta_{2^{n}}^{r_{k}}\right)}^{T}\delta_{2^{n}}^{r_{i}}=0,k\neq i$, we have


(3) $$\begin{array}{l} L\delta_{2^{n}}^{r_{i}}=\delta_{2^{n}}^{r_{i}}{\left(\delta_{2^{n}}^{r_{i}}\right)}^{T}\delta_{2^{n}}^{r_{i}}=\delta_{2^{n}}^{r_{i}}. \end{array}$$

Because of the arbitrariness of selecting *Y*^*i*^, Equation 3 implies that all modes *Y*^*k*^ are memorized and recognized by *L*. The proof is completed.

For convenience, we denote *Row*_*i*_(*Col*_*j*_(*L*)) by *l*_*ij*_. It is easy to observe that


(4) $$l_{ij}=\{\begin{array}{c} 1,\text{ if }i=j=r_{k},\\ 0,\text{ otherwise}. \end{array}$$

**Figure 1:**
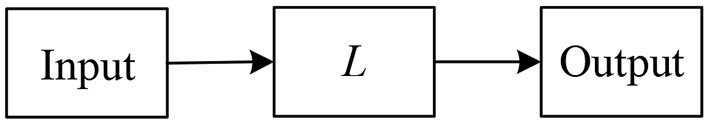
Recognition process of the memory model (Equation 2) proposed in the study.

From Equation 4, we find: (1) the memory matrix *L* is a Boolean matrix, (2) only on the main diagonal, there may be non-zero elements. For any mode $\delta_{2^{n}}^{r_{i}}$ to be recognized, if $L\delta_{2^{n}}^{r_{i}}=\text{0}$, this mode is not a stored standard learning mode. Otherwise, $L\delta_{2^{n}}^{r_{i}}$ must be $\delta_{2^{n}}^{r_{i}}$, which means the mode $\delta_{2^{n}}^{r_{i}}$ can be recognized accurately. The recognition process is shown in Figure 1.

Example 2. The four learning modes in Example 1 are used to verify the memory ability of the new memory model (Equation 2).

First, it is easy to calculate algebraic forms of the four learning modes as follows:


(5) $$\begin{array}{l} \begin{array}{c} Y^{1}={\left(1,1,0,1,1\right)}^{T}\sim \delta_{2}^{1}\delta_{2}^{1}\delta_{2}^{2}\delta_{2}^{1}\delta_{2}^{1}=\delta_{32}^{5},\\ Y^{2}={\left(1,0,1,0,0\right)}^{T}\sim \delta_{2}^{1}\delta_{2}^{2}\delta_{2}^{1}\delta_{2}^{2}\delta_{2}^{2}=\delta_{32}^{12},\\ Y^{3}={\left(0,1,1,0,1\right)}^{T}\sim \delta_{2}^{2}\delta_{2}^{1}\delta_{2}^{1}\delta_{2}^{2}\delta_{2}^{1}=\delta_{32}^{19},\\ Y^{4}={\left(0,0,0,1,1\right)}^{T}\sim \delta_{2}^{2}\delta_{2}^{2}\delta_{2}^{2}\delta_{2}^{1}\delta_{2}^{1}=\delta_{32}^{29}. \end{array} \end{array}$$

According to Equation 2, the memory matrix *L* is as follows:


(6) $$\begin{array}{l} \begin{array}{l} L=\delta_{32}^{5}{\left(\delta_{32}^{5}\right)}^{T}+\delta_{32}^{12}{\left(\delta_{32}^{12}\right)}^{T}+\delta_{32}^{19}{\left(\delta_{32}^{19}\right)}^{T}+\delta_{32}^{29}{\left(\delta_{32}^{29}\right)}^{T}\\ =\left[\begin{array}{cccccccccccccccccccccccccccccccc} 0 & 0 & 0 & 0 & 0 & 0 & 0 & 0 & 0 & 0 & 0 & 0 & 0 & 0 & 0 & 0 & 0 & 0 & 0 & 0 & 0 & 0 & 0 & 0 & 0 & 0 & 0 & 0 & 0 & 0 & 0 & 0\\ 0 & 0 & 0 & 0 & 0 & 0 & 0 & 0 & 0 & 0 & 0 & 0 & 0 & 0 & 0 & 0 & 0 & 0 & 0 & 0 & 0 & 0 & 0 & 0 & 0 & 0 & 0 & 0 & 0 & 0 & 0 & 0\\ 0 & 0 & 0 & 0 & 0 & 0 & 0 & 0 & 0 & 0 & 0 & 0 & 0 & 0 & 0 & 0 & 0 & 0 & 0 & 0 & 0 & 0 & 0 & 0 & 0 & 0 & 0 & 0 & 0 & 0 & 0 & 0\\ 0 & 0 & 0 & 0 & 0 & 0 & 0 & 0 & 0 & 0 & 0 & 0 & 0 & 0 & 0 & 0 & 0 & 0 & 0 & 0 & 0 & 0 & 0 & 0 & 0 & 0 & 0 & 0 & 0 & 0 & 0 & 0\\ 0 & 0 & 0 & 0 & 1 & 0 & 0 & 0 & 0 & 0 & 0 & 0 & 0 & 0 & 0 & 0 & 0 & 0 & 0 & 0 & 0 & 0 & 0 & 0 & 0 & 0 & 0 & 0 & 0 & 0 & 0 & 0\\ 0 & 0 & 0 & 0 & 0 & 0 & 0 & 0 & 0 & 0 & 0 & 0 & 0 & 0 & 0 & 0 & 0 & 0 & 0 & 0 & 0 & 0 & 0 & 0 & 0 & 0 & 0 & 0 & 0 & 0 & 0 & 0\\ 0 & 0 & 0 & 0 & 0 & 0 & 0 & 0 & 0 & 0 & 0 & 0 & 0 & 0 & 0 & 0 & 0 & 0 & 0 & 0 & 0 & 0 & 0 & 0 & 0 & 0 & 0 & 0 & 0 & 0 & 0 & 0\\ 0 & 0 & 0 & 0 & 0 & 0 & 0 & 0 & 0 & 0 & 0 & 0 & 0 & 0 & 0 & 0 & 0 & 0 & 0 & 0 & 0 & 0 & 0 & 0 & 0 & 0 & 0 & 0 & 0 & 0 & 0 & 0\\ 0 & 0 & 0 & 0 & 0 & 0 & 0 & 0 & 0 & 0 & 0 & 0 & 0 & 0 & 0 & 0 & 0 & 0 & 0 & 0 & 0 & 0 & 0 & 0 & 0 & 0 & 0 & 0 & 0 & 0 & 0 & 0\\ 0 & 0 & 0 & 0 & 0 & 0 & 0 & 0 & 0 & 0 & 0 & 0 & 0 & 0 & 0 & 0 & 0 & 0 & 0 & 0 & 0 & 0 & 0 & 0 & 0 & 0 & 0 & 0 & 0 & 0 & 0 & 0\\ 0 & 0 & 0 & 0 & 0 & 0 & 0 & 0 & 0 & 0 & 0 & 0 & 0 & 0 & 0 & 0 & 0 & 0 & 0 & 0 & 0 & 0 & 0 & 0 & 0 & 0 & 0 & 0 & 0 & 0 & 0 & 0\\ 0 & 0 & 0 & 0 & 0 & 0 & 0 & 0 & 0 & 0 & 0 & 1 & 0 & 0 & 0 & 0 & 0 & 0 & 0 & 0 & 0 & 0 & 0 & 0 & 0 & 0 & 0 & 0 & 0 & 0 & 0 & 0\\ 0 & 0 & 0 & 0 & 0 & 0 & 0 & 0 & 0 & 0 & 0 & 0 & 0 & 0 & 0 & 0 & 0 & 0 & 0 & 0 & 0 & 0 & 0 & 0 & 0 & 0 & 0 & 0 & 0 & 0 & 0 & 0\\ 0 & 0 & 0 & 0 & 0 & 0 & 0 & 0 & 0 & 0 & 0 & 0 & 0 & 0 & 0 & 0 & 0 & 0 & 0 & 0 & 0 & 0 & 0 & 0 & 0 & 0 & 0 & 0 & 0 & 0 & 0 & 0\\ 0 & 0 & 0 & 0 & 0 & 0 & 0 & 0 & 0 & 0 & 0 & 0 & 0 & 0 & 0 & 0 & 0 & 0 & 0 & 0 & 0 & 0 & 0 & 0 & 0 & 0 & 0 & 0 & 0 & 0 & 0 & 0\\ 0 & 0 & 0 & 0 & 0 & 0 & 0 & 0 & 0 & 0 & 0 & 0 & 0 & 0 & 0 & 0 & 0 & 0 & 0 & 0 & 0 & 0 & 0 & 0 & 0 & 0 & 0 & 0 & 0 & 0 & 0 & 0\\ 0 & 0 & 0 & 0 & 0 & 0 & 0 & 0 & 0 & 0 & 0 & 0 & 0 & 0 & 0 & 0 & 0 & 0 & 0 & 0 & 0 & 0 & 0 & 0 & 0 & 0 & 0 & 0 & 0 & 0 & 0 & 0\\ 0 & 0 & 0 & 0 & 0 & 0 & 0 & 0 & 0 & 0 & 0 & 0 & 0 & 0 & 0 & 0 & 0 & 0 & 0 & 0 & 0 & 0 & 0 & 0 & 0 & 0 & 0 & 0 & 0 & 0 & 0 & 0\\ 0 & 0 & 0 & 0 & 0 & 0 & 0 & 0 & 0 & 0 & 0 & 0 & 0 & 0 & 0 & 0 & 0 & 0 & 1 & 0 & 0 & 0 & 0 & 0 & 0 & 0 & 0 & 0 & 0 & 0 & 0 & 0\\ 0 & 0 & 0 & 0 & 0 & 0 & 0 & 0 & 0 & 0 & 0 & 0 & 0 & 0 & 0 & 0 & 0 & 0 & 0 & 0 & 0 & 0 & 0 & 0 & 0 & 0 & 0 & 0 & 0 & 0 & 0 & 0\\ 0 & 0 & 0 & 0 & 0 & 0 & 0 & 0 & 0 & 0 & 0 & 0 & 0 & 0 & 0 & 0 & 0 & 0 & 0 & 0 & 0 & 0 & 0 & 0 & 0 & 0 & 0 & 0 & 0 & 0 & 0 & 0\\ 0 & 0 & 0 & 0 & 0 & 0 & 0 & 0 & 0 & 0 & 0 & 0 & 0 & 0 & 0 & 0 & 0 & 0 & 0 & 0 & 0 & 0 & 0 & 0 & 0 & 0 & 0 & 0 & 0 & 0 & 0 & 0\\ 0 & 0 & 0 & 0 & 0 & 0 & 0 & 0 & 0 & 0 & 0 & 0 & 0 & 0 & 0 & 0 & 0 & 0 & 0 & 0 & 0 & 0 & 0 & 0 & 0 & 0 & 0 & 0 & 0 & 0 & 0 & 0\\ 0 & 0 & 0 & 0 & 0 & 0 & 0 & 0 & 0 & 0 & 0 & 0 & 0 & 0 & 0 & 0 & 0 & 0 & 0 & 0 & 0 & 0 & 0 & 0 & 0 & 0 & 0 & 0 & 0 & 0 & 0 & 0\\ 0 & 0 & 0 & 0 & 0 & 0 & 0 & 0 & 0 & 0 & 0 & 0 & 0 & 0 & 0 & 0 & 0 & 0 & 0 & 0 & 0 & 0 & 0 & 0 & 0 & 0 & 0 & 0 & 0 & 0 & 0 & 0\\ 0 & 0 & 0 & 0 & 0 & 0 & 0 & 0 & 0 & 0 & 0 & 0 & 0 & 0 & 0 & 0 & 0 & 0 & 0 & 0 & 0 & 0 & 0 & 0 & 0 & 0 & 0 & 0 & 0 & 0 & 0 & 0\\ 0 & 0 & 0 & 0 & 0 & 0 & 0 & 0 & 0 & 0 & 0 & 0 & 0 & 0 & 0 & 0 & 0 & 0 & 0 & 0 & 0 & 0 & 0 & 0 & 0 & 0 & 0 & 0 & 0 & 0 & 0 & 0\\ 0 & 0 & 0 & 0 & 0 & 0 & 0 & 0 & 0 & 0 & 0 & 0 & 0 & 0 & 0 & 0 & 0 & 0 & 0 & 0 & 0 & 0 & 0 & 0 & 0 & 0 & 0 & 0 & 0 & 0 & 0 & 0\\ 0 & 0 & 0 & 0 & 0 & 0 & 0 & 0 & 0 & 0 & 0 & 0 & 0 & 0 & 0 & 0 & 0 & 0 & 0 & 0 & 0 & 0 & 0 & 0 & 0 & 0 & 0 & 0 & 1 & 0 & 0 & 0\\ 0 & 0 & 0 & 0 & 0 & 0 & 0 & 0 & 0 & 0 & 0 & 0 & 0 & 0 & 0 & 0 & 0 & 0 & 0 & 0 & 0 & 0 & 0 & 0 & 0 & 0 & 0 & 0 & 0 & 0 & 0 & 0\\ 0 & 0 & 0 & 0 & 0 & 0 & 0 & 0 & 0 & 0 & 0 & 0 & 0 & 0 & 0 & 0 & 0 & 0 & 0 & 0 & 0 & 0 & 0 & 0 & 0 & 0 & 0 & 0 & 0 & 0 & 0 & 0\\ 0 & 0 & 0 & 0 & 0 & 0 & 0 & 0 & 0 & 0 & 0 & 0 & 0 & 0 & 0 & 0 & 0 & 0 & 0 & 0 & 0 & 0 & 0 & 0 & 0 & 0 & 0 & 0 & 0 & 0 & 0 & 0 \end{array}\right].. \end{array} \end{array}$$

Next, we verify the memory ability of *L*.


(7) $$\begin{array}{l} \begin{array}{l} LY^{1}=\left[\delta_{32}^{5}{\left(\delta_{32}^{5}\right)}^{T}+\delta_{32}^{12}{\left(\delta_{32}^{12}\right)}^{T}+\delta_{32}^{19}{\left(\delta_{32}^{19}\right)}^{T}+\delta_{32}^{29}{\left(\delta_{32}^{29}\right)}^{T}\right]\delta_{32}^{5}=\delta_{32}^{5}\\ \;=Y^{1},\\ LY^{2}=\left[\delta_{32}^{5}{\left(\delta_{32}^{5}\right)}^{T}+\delta_{32}^{12}{\left(\delta_{32}^{12}\right)}^{T}+\delta_{32}^{19}{\left(\delta_{32}^{19}\right)}^{T}+\delta_{32}^{29}{\left(\delta_{32}^{29}\right)}^{T}\right]\delta_{32}^{12}=\delta_{32}^{12}\\ \;=Y^{2},\\ LY^{3}=\left[\delta_{32}^{5}{\left(\delta_{32}^{5}\right)}^{T}+\delta_{32}^{12}{\left(\delta_{32}^{12}\right)}^{T}+\delta_{32}^{19}{\left(\delta_{32}^{19}\right)}^{T}+\delta_{32}^{29}{\left(\delta_{32}^{29}\right)}^{T}\right]\delta_{32}^{19}=\delta_{32}^{19}\\ \;=Y^{3},\\ LY^{4}=\left[\delta_{32}^{5}{\left(\delta_{32}^{5}\right)}^{T}+\delta_{32}^{12}{\left(\delta_{32}^{12}\right)}^{T}+\delta_{32}^{19}{\left(\delta_{32}^{19}\right)}^{T}+\delta_{32}^{29}{\left(\delta_{32}^{29}\right)}^{T}\right]\delta_{32}^{29}=\delta_{32}^{29}\\ \;=Y^{4}. \end{array} \end{array}$$

Remark 1. From the verification results in Equation 7, it is found that the four learning modes in Example 1 can be completely remembered by our memory model (Equation 6) without any errors. Furthermore, it can be directly verified that all modes from $\Delta_{2^{n}}$ can be accurately remembered if learned by our model.

Although this new memory model (Equation 2) can remember all modes accurately, its associative ability (robustness) is not good. Especially, in the real environment, there always be interference signals that may cause bit errors and then lead to recognition error. Therefore, it is necessary to improve the associative ability of the memory model.

### 3.2 Update the associative memory matrix *L*

As mentioned above, there are always interference signals that cause some bit errors in a realistic environment. When the error is within the allowable range, the interfered mode should be correctly identified. However, the memory model (Equation 2) cannot achieve this goal because it lacks associative ability.

In practical engineering, the accuracy of mode recognition is often guaranteed through an error control parameter. There are many such parameters. The bit error control parameter (BEC) is the simplest and most effective one among them. Specifically, the number of different bits between the input mode *i* and the *k*th stored learning mode, denoted by *s*_*ki*_, is used to define the degree of difference between the two modes. If *s*_*ki*_ is smaller than or equal to BEC, mode *i* may be recognized and classified as the *k*th learning mode. Otherwise, it does not belong to the *k*th learning mode. One thing to be considered is that if there are multiple learning modes with difference degrees less than or equal to the BEC, mode *i* should be classified into the above learning modes according to a probability distribution.

Without loss of generality, we let mode *i* be different from all learning modes that have been stored in the associative memory matrix *L*. In the following, we provide an update algorithm for *L* in Algorithm 2, according to a given BEC.

**Algorithm 2:** The associative memory matrix *L* in Equation 2 can be updated according to a given BEC to improve its associative ability.

1:	Determine a BEC *E*_*c*_ according to engineering requirements. Calculate the number of
	different bits between a mode *i* to be recognized and the *k*th stored learning mode,
	and denote the number by *s*_*ki*_(*k* = 1, 2, ⋯ , *m*).
2:	for *k* = 1 to *m* do
3:	(Calculate the transition probability *l*_*r*_*k*_*r*_*i*__.)
4:	if 0 < *s*_*ki*_ ≤ *E*_*c*_, then
5:	$l_{r_{k}r_{i}}=\frac{\frac{1}{s_{ki}}}{\underset{0<s_{ji}\leq E_{c}}{\overset{m}{\underset{j=1}{\sum }}}\frac{1}{s_{ji}}}$;
6:	else
7:	*l*_*r*_*k*_*r*_*i*__ = 0.
8:	end if
9:	end for
10:	Return the updated associative memory matrix $L={\left(l_{r_{k}r_{i}}\right)}_{2^{n}\times 2^{n}}$.

It is worth noting that the parameter *s*_*ki*_ indicates the difference degree between mode *i* and mode *k*. The larger *s*_*ki*_ is, the greater the difference between the two modes is and the lower the probability of mode *i* being classified as the *k*th learning mode is.

### 3.3 Learning and association of the new associative memory model

We put the results of Sections 3.1, 3.2 together and then present a new algorithm for the learning and association of model (Equation 2).

**Figure 2:**
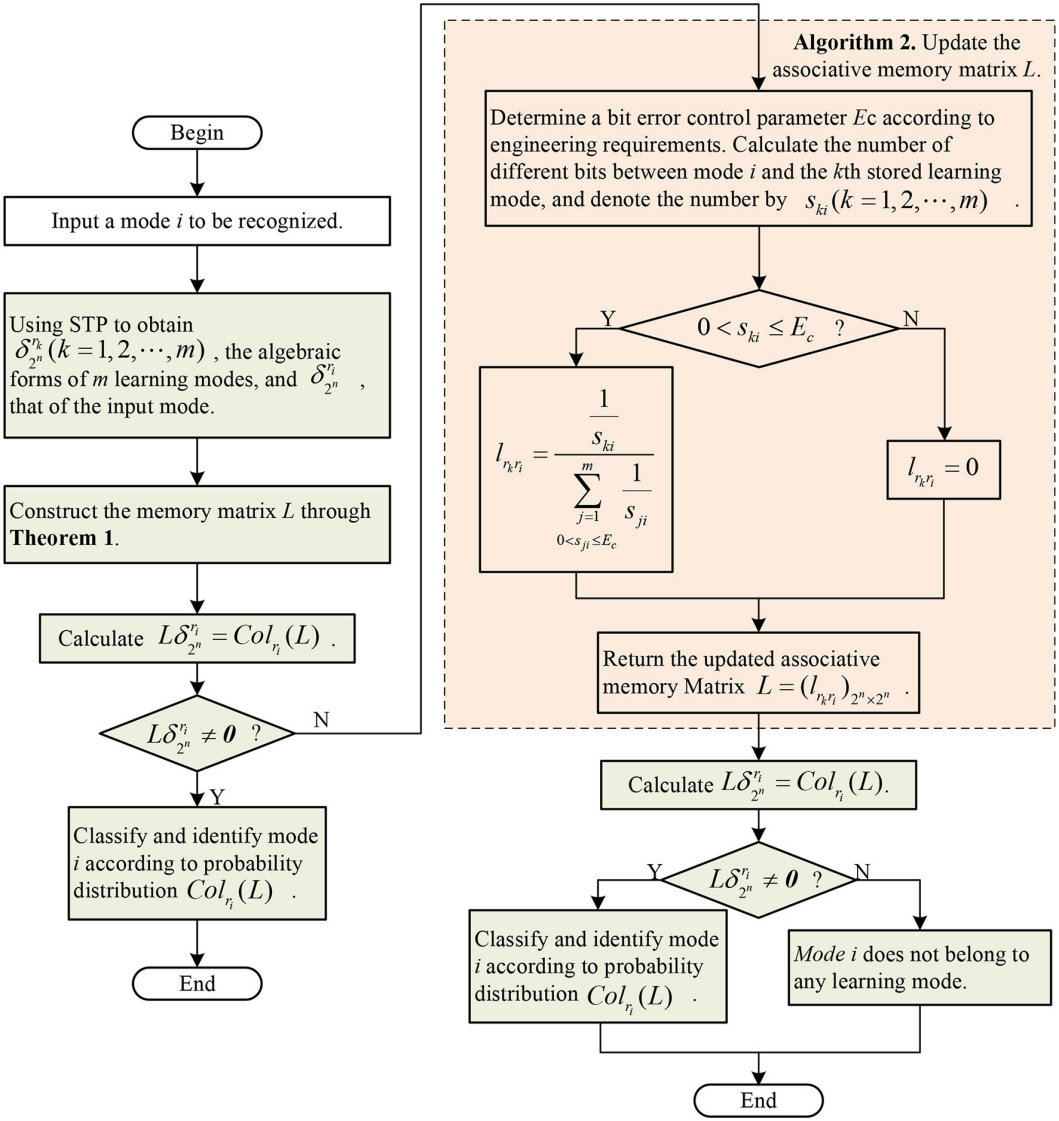
Flowchart for the novel associative memory model based on STP.

For ease of understanding, the use and learning process of our model are illustrated in the form of a flowchart, see Figure 2.

**Figure 3:**
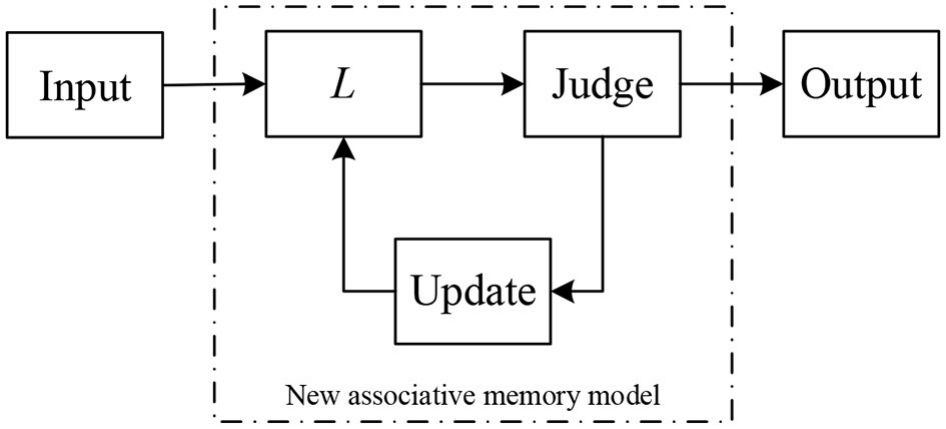
The diagram for the associative memory model proposed in this study.

Algorithm 3 includes two core parts: recognition and learning. In fact, after an object to be recognized enters our model for recognition, if it is recognized as a standard mode, the model outputs the result. Otherwise, the model learns immediately, re-identifies the aforementioned object, and finally outputs the result, which is the final result. The above algorithm can be summarized as a diagram, see Figure 3.

**Algorithm 3:** Assume that there are *m* *n*-dimensional learning modes. The mode recognition and model learning can be carried out by the following steps.

*Step 1. Construct the memory matrix L according to Equation 2*.
*Step 2. For a mode* *i* *to be recognized, obtain its algebraic form* $\delta_{2^{n}}^{r_{i}}$.
*Step 3. Calculate* $L\delta_{2^{n}}^{r_{i}}=Col_{r_{i}}\left(L\right)$. *If* $L\delta_{2^{n}}^{r_{i}}\neq \text{0}$, *classify mode* *i* *according to the probability distribution* *Col*_*r*_*i*__(*L*). *Else, go to Step 4*.
*Step 4. Use Algorithm 2 to update the associative memory matrix* *L*.
*Step 5. Check whether* $L\delta_{2^{n}}^{r_{i}}\neq \text{0}$ *holds or not. If yes, classify mode* *i* *according to the probability distribution* *Col*_*r*_*i*__(*L*). *Else, Mode* *i* *does not belong to any learning mode*.

Compared with DHNN, this new associative memory model has the following advantages:

- It can accurately remember more learning modes with fewer nodes. All modes in their algebraic forms are orthogonal to each other. Therefore, theoretically speaking, our model can remember all modes.
- The accuracy of mode recognition can be effectively controlled. As analyzed in Section 3.2, the parameter BEC can be used to control the mode recognition errors.
- This model works more efficiently because it learns automatically and only when needed. That is, when the input mode cannot be recognized for the first time, our associative learning model automatically uses the above input mode to learn (update the memory matrix) and then proceeds re-recognition. This is different from the most existing models, which perform mode recognition after learning.

## 4 Discussion

In the following, an example is given to show the effectiveness of the method developed in this study.

Example 3. Use Algorithm 3 to verify the associative memory ability of the four learning modes in Example 1. Assume that the BEC is *E*_*c*_ = 2.

When the mode *X*^*i*^ to be recognized is one of the learning modes, it has been verified in Example 2 that *X*^*i*^ can be accurately identified. In the following, we mainly consider the case that the object to be recognized is not a stored learning mode.

Step 1. From Example 2, we know that the algebraic forms of four learning modes and the memory matrix *L* have been given in Equations 5, 6, respectively.

Step 2. Let the object to be recognized be


$$Y^{i}={\left(0,1,0,1,1\right)}^{T}\sim \delta_{2}^{2}\delta_{2}^{1}\delta_{2}^{2}\delta_{2}^{1}\delta_{2}^{1}=\delta_{32}^{21}.$$

Step 3. We calculate


$$\begin{array}{l} L\delta_{32}^{21}=Col_{21}(L)\\ \;=[0,0,0,0,0,0,0,0,0,0,0,0,0,0,0,0,0,0,0,0,0,0,\\ \;0,0,0,0,0,0,0,0,0,0{]}^{T}. \end{array}$$

Since $L\delta_{32}^{21}=\text{0}$, we should use Algorithm 2 to update the associative memory matrix *L*.

Step 4. Throughout calculation, we get the numbers of different bits between *Y*^*i*^ and *Y*^1^, *Y*^2^, *Y*^3^, and *Y*^4^ which are *s*_1*i*_ = 1, *s*_2*i*_ = 5, *s*_3*i*_ = 2, and *s*_4*i*_ = 1, respectively. It is noted that *E*_*c*_ = 2, *s*_1*i*_ ≤ *E*_*c*_, *s*_2*i*_ > *E*_*c*_, *s*_3*i*_ ≤ *E*_*c*_, *s*_4*i*_ ≤ *E*_*c*_. According to


$$l_{r_{k}r_{i}}=\{\begin{array}{cc} \frac{\frac{1}{s_{ki}}}{\sum_{\begin{array}{l} j=1\\ 0<s_{ji}\leq 2 \end{array}}^{4}\frac{1}{s_{ji}}}, & 0<s_{ki}\leq 2,\\ 0, & s_{ki}>2, \end{array}$$

we have $l_{5,21}=\frac{2}{5}$, *l*_12, 21_ = 0, $l_{19,21}=\frac{1}{5}$, and $l_{29,21}=\frac{2}{5}$.

Then, the 21th column of the associative memory matrix *L* is updated to

$Col_{21}(L)={\left[0,0,0,0,\frac{2}{5},0,0,0,0,0,0,0,0,0,0,0,0,0,\frac{1}{5},0,0,0,0,0,0,0,0,0,\frac{2}{5},0,0,0]\right]}^{T}$.

Step 5.


$$\begin{array}{l} L\delta_{32}^{21}=Col_{21}(L)\\ \;=[0,0,0,0,\frac{2}{5},0,0,0,0,0,0,0,0,0,0,0,0,0,\frac{1}{5},0,0,\\ \;0,0,0,0,0,0,0,\frac{2}{5},0,0,0{]}^{T}\\ \;=\frac{2}{5}[0,0,0,0,1,0,0,0,0,0,0,0,0,0,0,0,0,0,0,0,0,0,0,0,0,0,\\ \;0,0,0,0,0,0{]}^{T}\\ \;+\frac{1}{5}[0,0,0,0,0,0,0,0,0,0,0,0,0,0,0,0,0,0,1,0,0,0,0,\\ \;0,0,0,0,0,0,0,0,0{]}^{T}\\ \;+\frac{2}{5}[0,0,0,0,0,0,0,0,0,0,0,0,0,0,0,0,0,0,0,0,0,0,0,0,\\ \;0,0,0,0,1,0,0,0{]}^{T}\\ \;=\frac{2}{5}\delta_{32}^{5}+\frac{1}{5}\delta_{32}^{19}+\frac{2}{5}\delta_{32}^{29}. \end{array}$$

**Figure 4:**
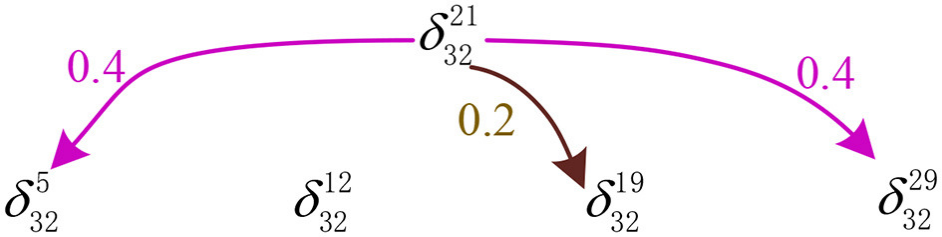
The recognition result of our model for the mode *X*^*i*^= (−1,1,−1,1,1).

Moreover, mode $\delta_{32}^{21}$ (equivalently, (0, 1, 0, 1, 1)^*T*^) is classified as $\delta_{32}^{5}$ (equivalently, (1, 1, 0, 1, 1)^*T*^), $\delta_{32}^{19}$ (equivalently, (0, 1, 1, 0, 1)^*T*^), and $\delta_{32}^{29}$ (equivalently, (0, 0, 0, 1, 1)^*T*^) stored in the network with probabilities of 0.4, 0.2, and 0.4, respectively. The recognition result of our model is shown in Figure 4.

From the example, we can observe that the associative memory can also be performed through Algorithm 3, even though the mode *X*^*i*^ is not one of the learning modes.

**Figure 5:**
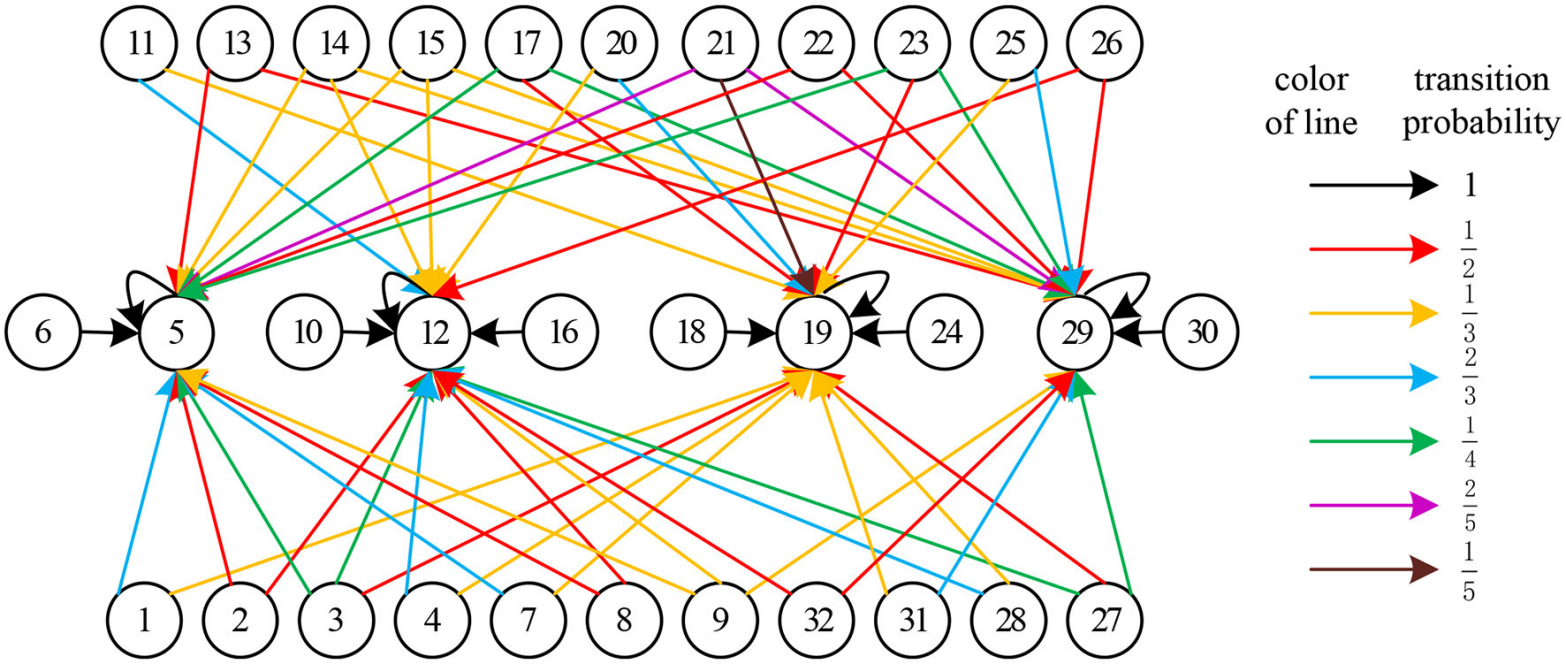
State transition diagram of matrix *L*.

If all modes in $\Delta_{2^{5}}$ are identified, the leaning result of *L* is as follows:


$$L=\left[\begin{array}{cccccccccccccccccccccccccccccccc} 0 & 0 & 0 & 0 & 0 & 0 & 0 & 0 & 0 & 0 & 0 & 0 & 0 & 0 & 0 & 0 & 0 & 0 & 0 & 0 & 0 & 0 & 0 & 0 & 0 & 0 & 0 & 0 & 0 & 0 & 0 & 0\\ 0 & 0 & 0 & 0 & 0 & 0 & 0 & 0 & 0 & 0 & 0 & 0 & 0 & 0 & 0 & 0 & 0 & 0 & 0 & 0 & 0 & 0 & 0 & 0 & 0 & 0 & 0 & 0 & 0 & 0 & 0 & 0\\ 0 & 0 & 0 & 0 & 0 & 0 & 0 & 0 & 0 & 0 & 0 & 0 & 0 & 0 & 0 & 0 & 0 & 0 & 0 & 0 & 0 & 0 & 0 & 0 & 0 & 0 & 0 & 0 & 0 & 0 & 0 & 0\\ 0 & 0 & 0 & 0 & 0 & 0 & 0 & 0 & 0 & 0 & 0 & 0 & 0 & 0 & 0 & 0 & 0 & 0 & 0 & 0 & 0 & 0 & 0 & 0 & 0 & 0 & 0 & 0 & 0 & 0 & 0 & 0\\ \frac{2}{3} & \frac{1}{2} & \frac{1}{4} & 0 & 1 & 1 & \frac{2}{3} & \frac{1}{2} & \frac{1}{3} & 0 & 0 & 0 & \frac{1}{2} & \frac{1}{3} & \frac{1}{3} & 0 & \frac{1}{4} & 0 & 0 & 0 & \frac{2}{5} & \frac{1}{2} & \frac{1}{4} & 0 & 0 & 0 & 0 & 0 & 0 & 0 & 0 & 0\\ 0 & 0 & 0 & 0 & 0 & 0 & 0 & 0 & 0 & 0 & 0 & 0 & 0 & 0 & 0 & 0 & 0 & 0 & 0 & 0 & 0 & 0 & 0 & 0 & 0 & 0 & 0 & 0 & 0 & 0 & 0 & 0\\ 0 & 0 & 0 & 0 & 0 & 0 & 0 & 0 & 0 & 0 & 0 & 0 & 0 & 0 & 0 & 0 & 0 & 0 & 0 & 0 & 0 & 0 & 0 & 0 & 0 & 0 & 0 & 0 & 0 & 0 & 0 & 0\\ 0 & 0 & 0 & 0 & 0 & 0 & 0 & 0 & 0 & 0 & 0 & 0 & 0 & 0 & 0 & 0 & 0 & 0 & 0 & 0 & 0 & 0 & 0 & 0 & 0 & 0 & 0 & 0 & 0 & 0 & 0 & 0\\ 0 & 0 & 0 & 0 & 0 & 0 & 0 & 0 & 0 & 0 & 0 & 0 & 0 & 0 & 0 & 0 & 0 & 0 & 0 & 0 & 0 & 0 & 0 & 0 & 0 & 0 & 0 & 0 & 0 & 0 & 0 & 0\\ 0 & 0 & 0 & 0 & 0 & 0 & 0 & 0 & 0 & 0 & 0 & 0 & 0 & 0 & 0 & 0 & 0 & 0 & 0 & 0 & 0 & 0 & 0 & 0 & 0 & 0 & 0 & 0 & 0 & 0 & 0 & 0\\ 0 & 0 & 0 & 0 & 0 & 0 & 0 & 0 & 0 & 0 & 0 & 0 & 0 & 0 & 0 & 0 & 0 & 0 & 0 & 0 & 0 & 0 & 0 & 0 & 0 & 0 & 0 & 0 & 0 & 0 & 0 & 0\\ 0 & \frac{1}{2} & \frac{1}{4} & \frac{2}{3} & 0 & 0 & 0 & \frac{1}{2} & \frac{1}{3} & 1 & \frac{2}{3} & 1 & 0 & \frac{1}{3} & \frac{1}{3} & 1 & 0 & 0 & 0 & \frac{1}{3} & 0 & 0 & 0 & 0 & 0 & \frac{1}{2} & \frac{1}{4} & \frac{2}{3} & 0 & 0 & 0 & \frac{1}{2}\\ 0 & 0 & 0 & 0 & 0 & 0 & 0 & 0 & 0 & 0 & 0 & 0 & 0 & 0 & 0 & 0 & 0 & 0 & 0 & 0 & 0 & 0 & 0 & 0 & 0 & 0 & 0 & 0 & 0 & 0 & 0 & 0\\ 0 & 0 & 0 & 0 & 0 & 0 & 0 & 0 & 0 & 0 & 0 & 0 & 0 & 0 & 0 & 0 & 0 & 0 & 0 & 0 & 0 & 0 & 0 & 0 & 0 & 0 & 0 & 0 & 0 & 0 & 0 & 0\\ 0 & 0 & 0 & 0 & 0 & 0 & 0 & 0 & 0 & 0 & 0 & 0 & 0 & 0 & 0 & 0 & 0 & 0 & 0 & 0 & 0 & 0 & 0 & 0 & 0 & 0 & 0 & 0 & 0 & 0 & 0 & 0\\ 0 & 0 & 0 & 0 & 0 & 0 & 0 & 0 & 0 & 0 & 0 & 0 & 0 & 0 & 0 & 0 & 0 & 0 & 0 & 0 & 0 & 0 & 0 & 0 & 0 & 0 & 0 & 0 & 0 & 0 & 0 & 0\\ 0 & 0 & 0 & 0 & 0 & 0 & 0 & 0 & 0 & 0 & 0 & 0 & 0 & 0 & 0 & 0 & 0 & 0 & 0 & 0 & 0 & 0 & 0 & 0 & 0 & 0 & 0 & 0 & 0 & 0 & 0 & 0\\ 0 & 0 & 0 & 0 & 0 & 0 & 0 & 0 & 0 & 0 & 0 & 0 & 0 & 0 & 0 & 0 & 0 & 0 & 0 & 0 & 0 & 0 & 0 & 0 & 0 & 0 & 0 & 0 & 0 & 0 & 0 & 0\\ \frac{1}{3} & 0 & \frac{1}{2} & \frac{1}{3} & 0 & 0 & \frac{1}{3} & 0 & 0 & 0 & \frac{1}{3} & 0 & 0 & 0 & 0 & 0 & \frac{1}{2} & 1 & 1 & \frac{2}{3} & \frac{1}{5} & 0 & \frac{1}{2} & 1 & \frac{1}{3} & 0 & \frac{1}{2} & \frac{1}{3} & 0 & 0 & \frac{1}{3} & 0\\ 0 & 0 & 0 & 0 & 0 & 0 & 0 & 0 & 0 & 0 & 0 & 0 & 0 & 0 & 0 & 0 & 0 & 0 & 0 & 0 & 0 & 0 & 0 & 0 & 0 & 0 & 0 & 0 & 0 & 0 & 0 & 0\\ 0 & 0 & 0 & 0 & 0 & 0 & 0 & 0 & 0 & 0 & 0 & 0 & 0 & 0 & 0 & 0 & 0 & 0 & 0 & 0 & 0 & 0 & 0 & 0 & 0 & 0 & 0 & 0 & 0 & 0 & 0 & 0\\ 0 & 0 & 0 & 0 & 0 & 0 & 0 & 0 & 0 & 0 & 0 & 0 & 0 & 0 & 0 & 0 & 0 & 0 & 0 & 0 & 0 & 0 & 0 & 0 & 0 & 0 & 0 & 0 & 0 & 0 & 0 & 0\\ 0 & 0 & 0 & 0 & 0 & 0 & 0 & 0 & 0 & 0 & 0 & 0 & 0 & 0 & 0 & 0 & 0 & 0 & 0 & 0 & 0 & 0 & 0 & 0 & 0 & 0 & 0 & 0 & 0 & 0 & 0 & 0\\ 0 & 0 & 0 & 0 & 0 & 0 & 0 & 0 & 0 & 0 & 0 & 0 & 0 & 0 & 0 & 0 & 0 & 0 & 0 & 0 & 0 & 0 & 0 & 0 & 0 & 0 & 0 & 0 & 0 & 0 & 0 & 0\\ 0 & 0 & 0 & 0 & 0 & 0 & 0 & 0 & 0 & 0 & 0 & 0 & 0 & 0 & 0 & 0 & 0 & 0 & 0 & 0 & 0 & 0 & 0 & 0 & 0 & 0 & 0 & 0 & 0 & 0 & 0 & 0\\ 0 & 0 & 0 & 0 & 0 & 0 & 0 & 0 & 0 & 0 & 0 & 0 & 0 & 0 & 0 & 0 & 0 & 0 & 0 & 0 & 0 & 0 & 0 & 0 & 0 & 0 & 0 & 0 & 0 & 0 & 0 & 0\\ 0 & 0 & 0 & 0 & 0 & 0 & 0 & 0 & 0 & 0 & 0 & 0 & 0 & 0 & 0 & 0 & 0 & 0 & 0 & 0 & 0 & 0 & 0 & 0 & 0 & 0 & 0 & 0 & 0 & 0 & 0 & 0\\ 0 & 0 & 0 & 0 & 0 & 0 & 0 & 0 & 0 & 0 & 0 & 0 & 0 & 0 & 0 & 0 & 0 & 0 & 0 & 0 & 0 & 0 & 0 & 0 & 0 & 0 & 0 & 0 & 0 & 0 & 0 & 0\\ 0 & 0 & 0 & 0 & 0 & 0 & 0 & 0 & \frac{1}{3} & 0 & 0 & 0 & \frac{1}{2} & \frac{1}{3} & \frac{1}{3} & 0 & \frac{1}{4} & 0 & 0 & 0 & \frac{2}{5} & \frac{1}{2} & \frac{1}{4} & 0 & \frac{2}{3} & \frac{1}{2} & \frac{1}{4} & 0 & 1 & 1 & \frac{2}{3} & \frac{1}{2}\\ 0 & 0 & 0 & 0 & 0 & 0 & 0 & 0 & 0 & 0 & 0 & 0 & 0 & 0 & 0 & 0 & 0 & 0 & 0 & 0 & 0 & 0 & 0 & 0 & 0 & 0 & 0 & 0 & 0 & 0 & 0 & 0\\ 0 & 0 & 0 & 0 & 0 & 0 & 0 & 0 & 0 & 0 & 0 & 0 & 0 & 0 & 0 & 0 & 0 & 0 & 0 & 0 & 0 & 0 & 0 & 0 & 0 & 0 & 0 & 0 & 0 & 0 & 0 & 0\\ 0 & 0 & 0 & 0 & 0 & 0 & 0 & 0 & 0 & 0 & 0 & 0 & 0 & 0 & 0 & 0 & 0 & 0 & 0 & 0 & 0 & 0 & 0 & 0 & 0 & 0 & 0 & 0 & 0 & 0 & 0 & 0 \end{array}\right],$$

whose state transition diagram is shown in Figure 5.

Of course, in practical application, it is not necessary to calculate all elements of *L*. In fact, when mode *i* requires to be identified, we only need to determine the value of the *r*_*i*_th column of matrix *L*.

## 5 Conclusion

A new associative memory model has been proposed to solve the problem of information storage capacity. The main mathematical tool used in this study was STP. It can equivalently transform ordinary vector modes into the algebraic forms that are mutually orthogonal. Therefore, the model proposed in this study can accurately remember all learning modes theoretically. However, due to the complexity of the environment, there are inevitably some interfering factors. Hence, in order to improve the association ability or robustness of the model, an on-demand learning update algorithm for the model was developed. It utilizes parameter BEC to effectively control the recognition errors. Compared with the classical DHNN, the new model can store more information with fewer nodes.

However, when the parameter BEC is too large, the new model may encounter such a situation that the input mode belongs to multiple different learning modes, according to a probability distribution. In this case, the input mode may not be accurately recognized. Therefore, how to choose the best BEC based on the actual situation and how to apply the associative memory model to practical scenarios as soon as possible are our future research topics.

## Data availability statement

The original contributions presented in the study are included in the article/supplementary material, further inquiries can be directed to the corresponding author.

## Author contributions

YH: Conceptualization, Formal analysis, Investigation, Methodology, Validation, Writing—original draft, Writing—review & editing. HT: Conceptualization, Formal analysis, Funding acquisition, Methodology, Project administration, Supervision, Writing—review & editing. CW: Formal analysis, Investigation, Writing—review & editing.
